# The role of touch in regulating inter-partner physiological coupling during empathy for pain

**DOI:** 10.1038/s41598-017-03627-7

**Published:** 2017-06-12

**Authors:** Pavel Goldstein, Irit Weissman-Fogel, Simone G. Shamay-Tsoory

**Affiliations:** 1 Department of Psychology, University of Haifa, Haifa, Israel; 2 Department of Statistics, University of Haifa, Haifa, Israel; 3 The Emili Sagol Creative Arts Therapies Research Center, University of Haifa, Haifa, Israel; 4 Physical Therapy Department, Faculty of Social Welfare and Health Sciences, University of Haifa, Haifa, Israel

## Abstract

The human ability to synchronize with other individuals is critical for the development of social behavior. Recent research has shown that physiological inter-personal synchronization may underlie behavioral synchrony. Nevertheless, the factors that modulate physiological coupling are still largely unknown. Here we suggest that social touch and empathy for pain may enhance interpersonal physiological coupling. Twenty-two romantic couples were assigned the roles of target (pain receiver) and observer (pain observer) under pain/no-pain and touch/no-touch conditions, and their ECG and respiration rates were recorded. The results indicate that the partner touch increased interpersonal respiration coupling under both pain and no-pain conditions and increased heart rate coupling under pain conditions. In addition, physiological coupling was diminished by pain in the absence of the partner’s touch. Critically, we found that high partner’s empathy and high levels of analgesia enhanced coupling during the partner’s touch. Collectively, the evidence indicates that social touch increases interpersonal physiological coupling during pain. Furthermore, the effects of touch on cardio-respiratory inter-partner coupling may contribute to the analgesic effects of touch via the autonomic nervous system.

## Introduction

The human capacity for generating events in synchrony^1^ with other individuals has important evolutional significance. Behavioral synchrony is evident in the animal kingdom in various forms. Among them are synchronized periodic movements to create acoustic signals^2–4^, synchronous flashing among fireflies^5^, synchronized collective movements among predators while hunting^6^ and synchronized reactions to stressful and dangerous situations^6–8^. Humans also tend to coordinate their actions and imitate the postures or actions of others whether they are aware of this or not^1, 9, 10^. This ability develops early in life^11, 12^ and is crucial for social communication in general^13^ and for the development of infant and mother bonding in particular^14^. Furthermore, synchronized coordinated behaviors have also been noted in other social behavioral contexts, such as speech understanding^15^ or psychotherapy^16^. These studies indicate that social synchrony plays a major role in affiliative behaviors and in the development of social behavior.

Recently, an increasing number of studies have explored the physiological mechanisms that underlie social synchrony. These studies have shown that group synchrony is accompanied by cardiac rhythms that are synchronized between active participants and bystanders during collective rituals^17^ and people collectively watching emotional movies^18^. In addition, cardiac and respiratory synchronization was found to underlie interpersonal action coordination during choir singing^19^. Similarly, dual synchrony between romantic dyads was associated with cardiac and respiratory coupling during gazing and imitation tasks^20^ and even simply when the two members of the couple are together^20^, suggesting that the mere presence of one’s partner may trigger heart rate synchrony. Nonetheless, although synchrony has been reported in an abundance of social contexts, the conditions that facilitate synchrony remain unclear.

One condition that may increase synchrony is empathy for pain, a concept that describes our tendency to experience distress automatically when confronted with someone else’s pain^21^. Empathy for pain is associated with activity in pain neural networks^22, 23^, along with physiological responses such as increased skin conductance^24^ and increased heart rate^17, 25^. Since sharing the sufferer’s pain constitutes empathy for pain, inflicting pain to a target may increase the coupling between sufferer (target) and observer. In line with this speculation, Levenson and Gottman (1983) showed that distress situations enhance physiological coupling in romantic dyads^26^. Therefore, we hypothesized that empathy for pain would increase synchrony between the physiological responses of the target and those of the observer.

Another condition that may promote synchrony is touch. Interpersonal touch has important social and affective values^27–29^. Specifically, skin-to-skin touch contributes to the development of premature infants^30^, regulates their stress responses^31^, provides comfort and emotional well-being^32–34^ and has an analgesic effect^34^. Physiologically, interpersonal touch increases the coupling of electrodermal activity and pulse rate variability^35^ and modulates blood pressure reactivity to stress^36^ as well as reactivity to distress^37^. Therefore, our second hypothesis was that interpersonal touch would increase interactional physiological coupling.

Furthermore, it has been shown that touch moderates (1) the relationship between the observer’s trait empathy and the target’s analgesia; (2) inter-partner synchrony of pain rating; and (3) touch-related analgesia^38^. Moreover, it has been found that empathic accuracy, i.e., the extent to which the supporting partner accurately estimates the pain of the suffering person, is related to the sufferer’s pain perception^39^. Accordingly, we predicted that inter-dyad variability in level of physiological coupling while experiencing pain would be moderated by levels of trait empathy and empathic accuracy and by the analgesic effect of touch.

**Figure 1:**
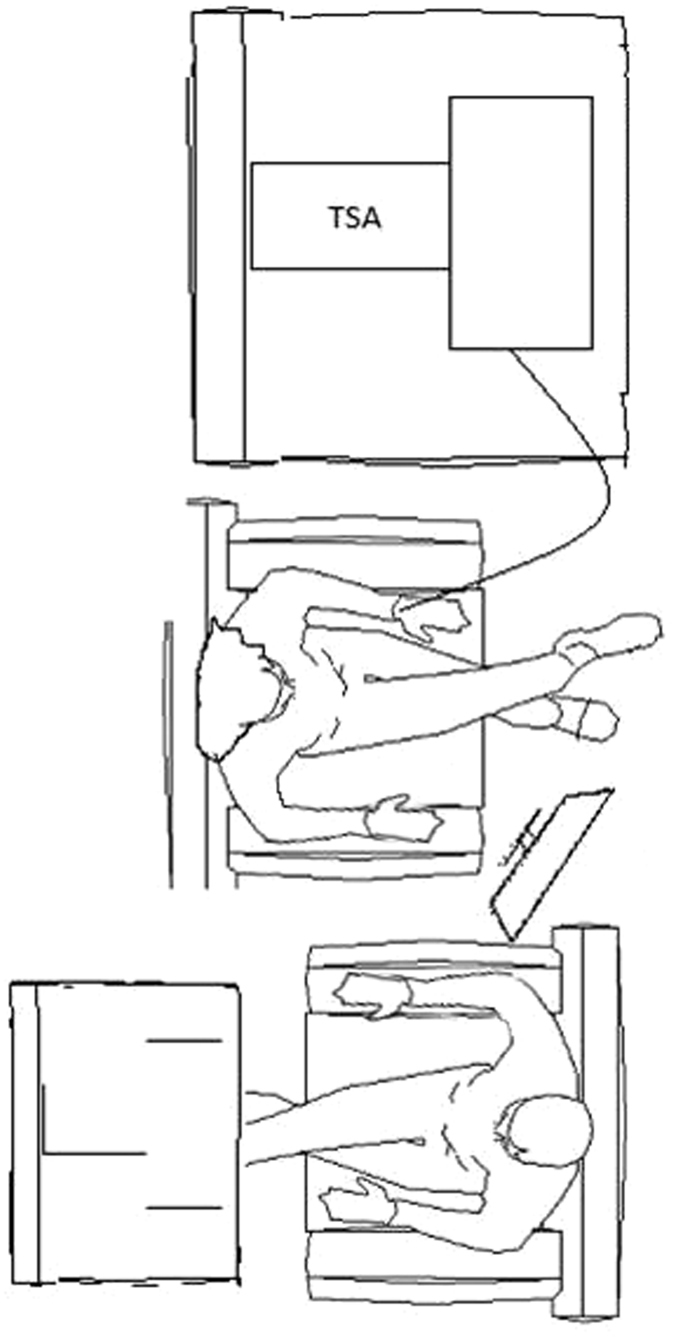
*No touch-pain condition.*

To examine these predictions, we designed an experiment consisting of six conditions in which romantic partners were instructed to hold hands or sit with no physical contact or in separated rooms during the pain vs. no pain conditions (Fig. 1). Throughout the experiment the electrocardiogram and respiration of both partners were simultaneously recorded.

## Results

The sample mean and standard deviations of the pain ratings of both partners appear in Table 1 (women rated their own pain and the men evaluated their partner’s pain). As was reported in the initial report of this data (Goldstein *et al*., 2016), the pain ratings in the *partner-touch* condition were lower than in the *partner-no touch* condition (M_diff_ = −0.36, p = 0.029) and the *pain-alone* condition (M_diff_ = −0.66, p < 0.001), confirming that touch had an analgesic effect. In addition, during the *pain-alone* condition, the women’s pain ratings were marginally higher than in the *partner-no touch* condition (M_diff_ = 0.29, p = 0.093).

**Table 1:** Average (standard deviation) pain ratings in each condition.

Condition	Woman	Partner^*^
pain-alone	52.41(29.41)	—
partner no-touch	37.74(24.82)	43.52(22.71)
partner touch	25.03(20.32)	38.51(17.32)

*The male partner guessed the woman’s pain.

### Respiration analysis

We analyzed the data using the coupled linear oscillator (CLO) model (see Methods section), estimating the inter-partner relationship between one partner’s inhalation (predictor) and the other partner’s exchange between inhalation and exhalation (outcome) in six combinations of pain (no pain/pain) and touch (alone/no-touch/touch) factors. The CLO analysis indicated that touch and pain moderated the partners’ velocity effect both in men and in women (F_(4,55000)_ = 14.44, p < 0.0001, ΔBIC = −47.8, ΔR^2^ = 0.18), indicating that the partner’s velocity effect differed across experimental conditions. We further carried out separate post-hoc analyses for men and women to examine differential effects in targets (women) and observers (men).

### Model for male participants: How changes in respiration in females predict shifts in changes of males

**Figure 2:**
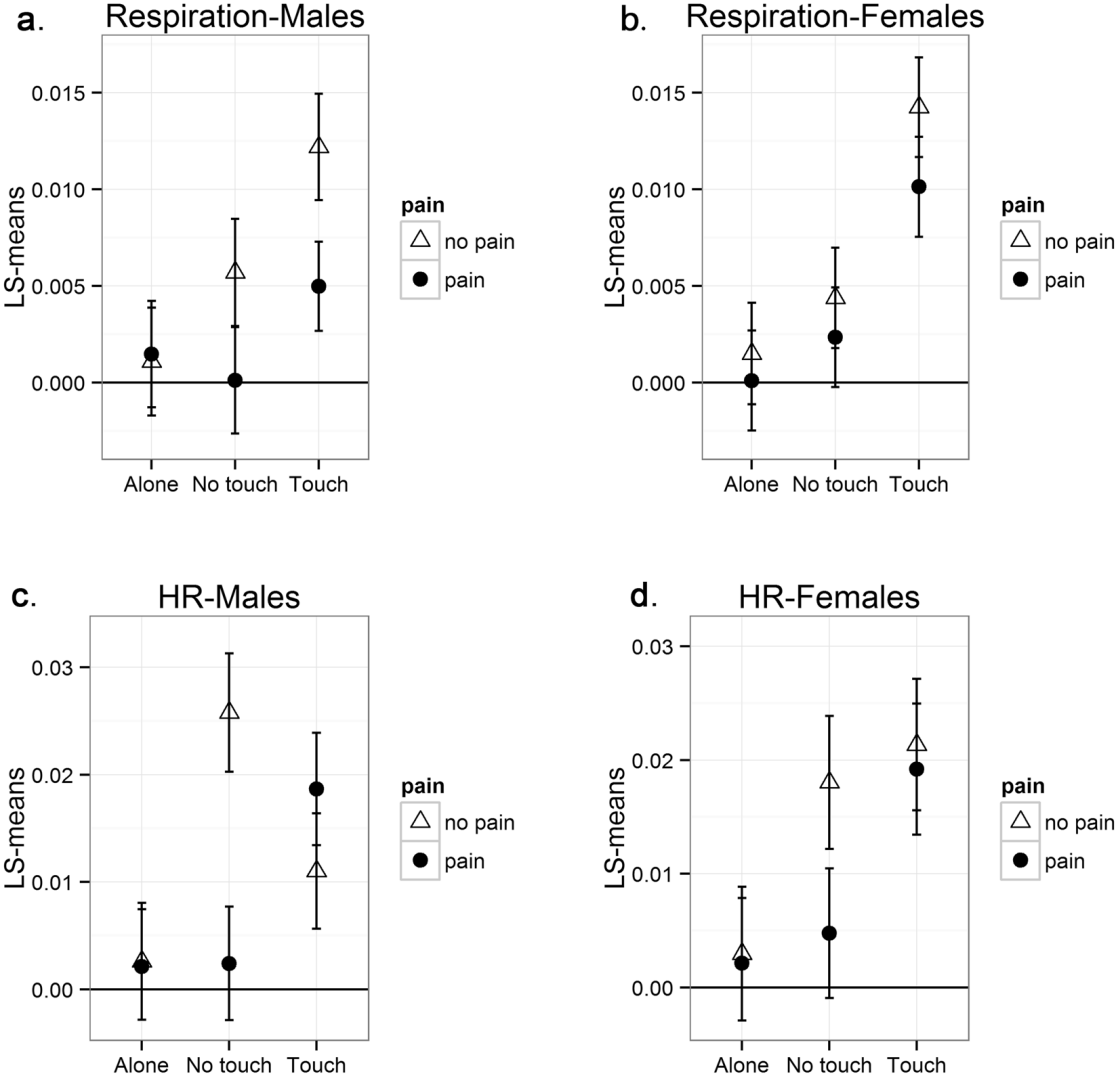
Results of the Coupled Linear Oscillator (CLO) Model for heart rate and respiration. For the sake of simplicity, results are presented as absolute values. The Y-axis presents models based on the least squares (LS) means of each experimental condition, expressing the level of physiological coupling in different experimental conditions. Zero represents a case without interpersonal coupling, while scores that differ from zero indicate interpersonal coupling.

**Figure 3:**
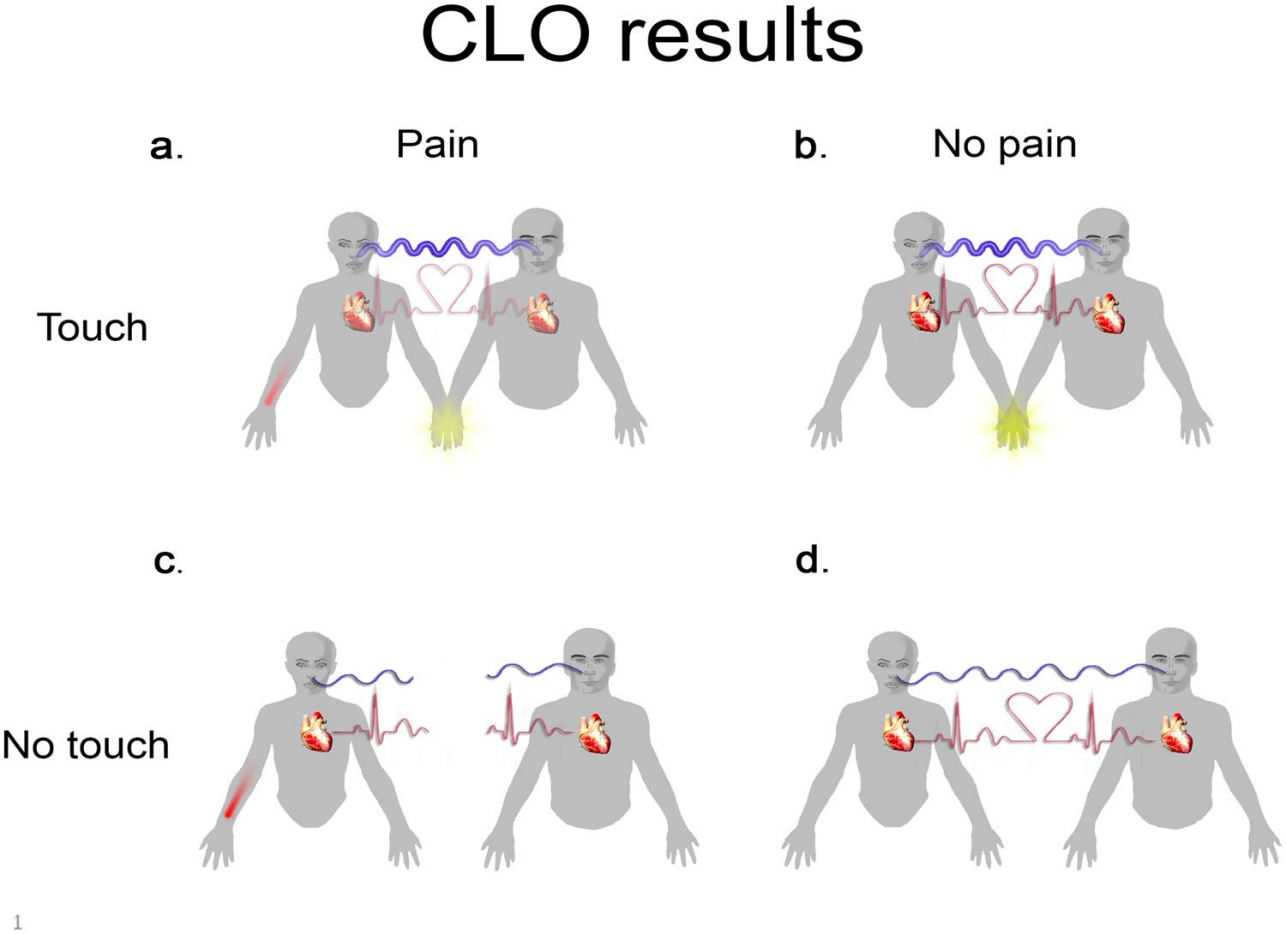
Graphical representation of Coupled Linear Oscillator (CLO) model findings for heart rate and respiration (Fig. 2). Blue lines represent respiration inter-partner coupling and red lines represent coupling in heart-rate. The line’s thickness represents the strength of the coupling, with broken lines denoting a total lack of the coupling. (**a**) Coupling of respiration and heart rate during *touch-pain* condition. (**b**) Coupling of respiration and heart rate during *touch-no pain* condition. (**c**) No coupling of respiration and heart rate during *no touch-pain* condition. (**d**) Coupling of respiration and heart rate during *no touch-no pain* condition.

The post-hoc analysis revealed a significant effect of cross-partner velocity for male participants during the *touch-no pain* (η_t(np)_ = −0.012, p < 0.001, 95% CI [−0.06, −0.18]), *no touch-no pain* (ζ_nt(np)_ = −0.006, p = 0.012, 95% CI [−0.001, −0.010]) and *touch-pain* (ζ_t(p)_ = −0.005, p = 0.017, 95% CI [−0.001, −0.010]) conditions (Fig. 2a). This pattern of effects describes a consistent pattern of inhalation among women while men shift from inhalation to exhalation. However, the woman’s cross-partner velocity during the *no touch-pain* condition (ζ_nt(p)_ < 0.001, p = 0.96) was not related to the man’s acceleration. No significant cross-partner effects were detected for the *pain alone* (ζ_a(p)_ = −0.001, p = 0.78) and the *no pain alone* (ζ_a(np)_ = 0.001, p = 0.86) conditions. In line with our hypotheses, the coupling during the *touch* conditions was higher than in the *no touch* conditions, whether without pain (Δζ_t/nt(np)_ = 0.006, p < 0.001, 95% CI [0.003, 0.008]) or with pain (Δζ_t/nt(p)_ = 0.012, p < 0.001, 95% CI [0.007, 0.016]). However, the pain vs. no pain comparison was associated with decreased respiration synchronization in both the *touch* (Δζ_t(p/np)_ = −0.007, p < 0.001, 95% CI [−0.004, 0.010]) and the *no touch* (Δζ_nt(p/np)_ = −0.006, p < 0.001, 95% CI [−0.004, 0.008]) conditions. Figure 3 depicts these findings.

### Model for female participants: How changes in respiration in males predict shifts in changes of females

In line with the male model, a significant effect of cross-partner velocity was found for women during the *touch-no pain* (ζ_t(np)_ = 0.014, p < 0.001, 95% CI [0.009, 0.019]) and the *touch-pain* (ζ_t(p)_ = 0.010, p < 0.001, 95% CI [0.006, 0.014]) conditions, while a marginal effect was found in the *no touch-no pain* condition (ζ_nt(np)_ = 0.004, p = 0.092, 95% CI [−0.001, 0.008]) (Fig. 2b). These effects indicated that women tend to shift from exhalation to inhalation when men inhale. However, women’s cross-partner velocity during the *no touch-pain condition* was not significant (ζ_nt(p)_ = 0.002, p = 0.54). No significant cross-partner effects were detected for the *pain alone* (ζ_a(p)_ = −0.001, p = 0.64) or the *no pain alone* (ζ_a(np)_ < 0.001, p = 0.92) conditions.

In line with our hypotheses, the coupling during *touch* increased compared to during *no touch* in both the *no pain* (Δζ_t/nt(np)_ = 0.010, p < 0.001, 95% CI [0.006, 0.014]) and the *pain* (Δζ_t/nt(p)_ = 0.008, p < 0.001, 95% CI [0.003, 0.012]) conditions. However, there was no difference between *pain* and *no pain* during both *touch* (Δζ_t(p/np)_ = 0.004, p = 0.115) and *no touch* (Δζ_nt(p/np)_ = 0.002, p = 0.637) conditions.

### Heart rate analysis

For heart rate we carried out a similar analysis based on the CLO model (see Methods section), estimating the inter-partner relationship between an increase in heart rate of one partner and the exchange between increase and decrease of heart rate in the second partner as a function of pain and touch factors. As in the case of respiratory rate, touch and pain moderated the partners’ velocity effect both in women and in men (F_(4,25000)_ = 19.40, p < 0.0001, −ΔBIC = 7.7, ΔR^2^ = 0.12), indicating that the partner velocity effect differed across experiment conditions. We further carried out separate post-hoc analyses for male and female participants.

### Model for male participants: How changes in respiration in females predict shifts in changes of males

The post-hoc analysis revealed a significant effect of cross-partner velocity for men during the *no touch-no pain* (ζ_nt(np)_ = 0.026, p < 0.001, 95% CI [0.016, 0.036]), *touch-pain* (ζ_t(p)_ = 0.019, p < 0.001, 95% CI [0.010, 0.027]) and *touch-no pain* (ζ_t(np)_ = 0.011, p < 0.001, 95% CI [0.005, 0.016]) conditions (Fig. 2c). Thus, an increase in the woman’s heart rate was related to a shift from a decrease to an increase in the man’s heart rate under the above-mentioned conditions. However, cross-partner female velocity during the *no touch-pain* condition (ζ_nt(p)_ = 0.002, p = 0.57) was not related to acceleration in the men’s heart rate. No significant cross-partner effects were detected for the *pain alone* (ζ_a(p)_ = −0.002, p = 0.62) and *no pain alone* (ζ_a(np)_ = 0.002, p = 0.56) conditions. In line with our hypotheses, the synchronization during *touch-pain* was higher than in the *no touch-pain* (Δζ_t/nt(p)_ = 0.017, p < 0.001, 95% CI [0.009, 0.024]) and the *touch-no pain* (Δζ_t(p/np)_ = 0.007, p < 0.001, 95% CI [0.003, 0.010]) conditions. However, during the *no touch-no pain* condition, there was increased synchronization compared to the *touch-no pain* (Δζ_t/nt(np)_ = 0.015, p < 0.001, 95% CI [0.008, 0.021]) and *no touch-pain* (Δζn_t(p/np)_ = 0.024, p < 0.001, 95% CI [0.014, 0.034]) conditions.

### Model for female participants: How changes in respiration in males predict shifts in changes of females

In line with the results of the model for male participants, a significant effect of cross-partner velocity was found for female participants during the *touch-no pain* (ζ_t(np)_ = 0.021, p < 0.001, 95% CI [0.012, 0.029]), *touch-pain* (ζ_t(p)_ = 0.019, p < 0.001, 95% CI [0.011, 0.027]) and *no touch-no pain* (ζ_nt(np)_ = 0.018, p < 0.001, 95% CI [0.010, 0.026]) conditions (Fig. 2d). These effects indicate that the increase in the men’s heart rate was associated with the change in the women’s heart rate from decreasing to increasing. In addition, the women’s cross-partner velocity was not related to the men’s acceleration in heart rate (ζ_nt(p)_ = −0.005, p = 0.32) in the *no touch-pain* (ζ_nt(p)_ = 0.004, p = 0.183), *pain alone* (ζ_a(p)_ = 0.002, p = 0.59) or the *no pain alone* (ζ_a(np)_ = 0.002, p = 0.71) conditions. The increased synchronization during the *touch-pain* condition compared to the *no touch-pain* (Δζ_t/nt(p)_ = 0.014, p < 0.001, 95% CI [0.007, 0.021]) and *touch-no pain* (Δζ_t(p/np)_ = 0.007, p < 0.001, 95% CI [0.003, 0.010]) conditions is in line with our hypothesis. However, the heart rate synchronization during the *touch-no pain* condition did not differ from the *touch-pain* (Δζ_t (np/p)_ = 0.002, p = 0.368) or the *no touch-no pain* (Δζn_t/nt(np)_ = 0.003, p = 0.274) conditions.

In summary, all four analyses (men/women X respiration/heart rate) followed a common pattern—touch increased synchronization during pain.

### Moderation analysis

We applied Confirmatory Factor Analysis (CFA) to test the structure of the Interpersonal Reactivity Index (IRI) questionnaire, assuming the same unique latent empathy content for both partners. The analysis revealed a good fit between the measurement model and the data (χ2/df = 1.69, CFI = 0.94, RMSEA = 0.071). Thus, in the following analysis we treated trait empathy as a single factor. For the empathy trait measure we used the average of all questions from the IRI questionnaire. Empathic accuracy and trait empathy measurements demonstrated high correlation (r = 0.62, p < 0.001). We tested the moderation effect of empathic accuracy, trait empathy and women’s analgesia on across-partner coupling of heart rate and respiration fluctuations in the *touch-pain* and *no touch pain* conditions. This analysis tested the hypothesis that the observer’s level of empathy, his empathic accuracy and the levels of pain analgesia moderate touch-related physiological coupling.

**Figure 4:**
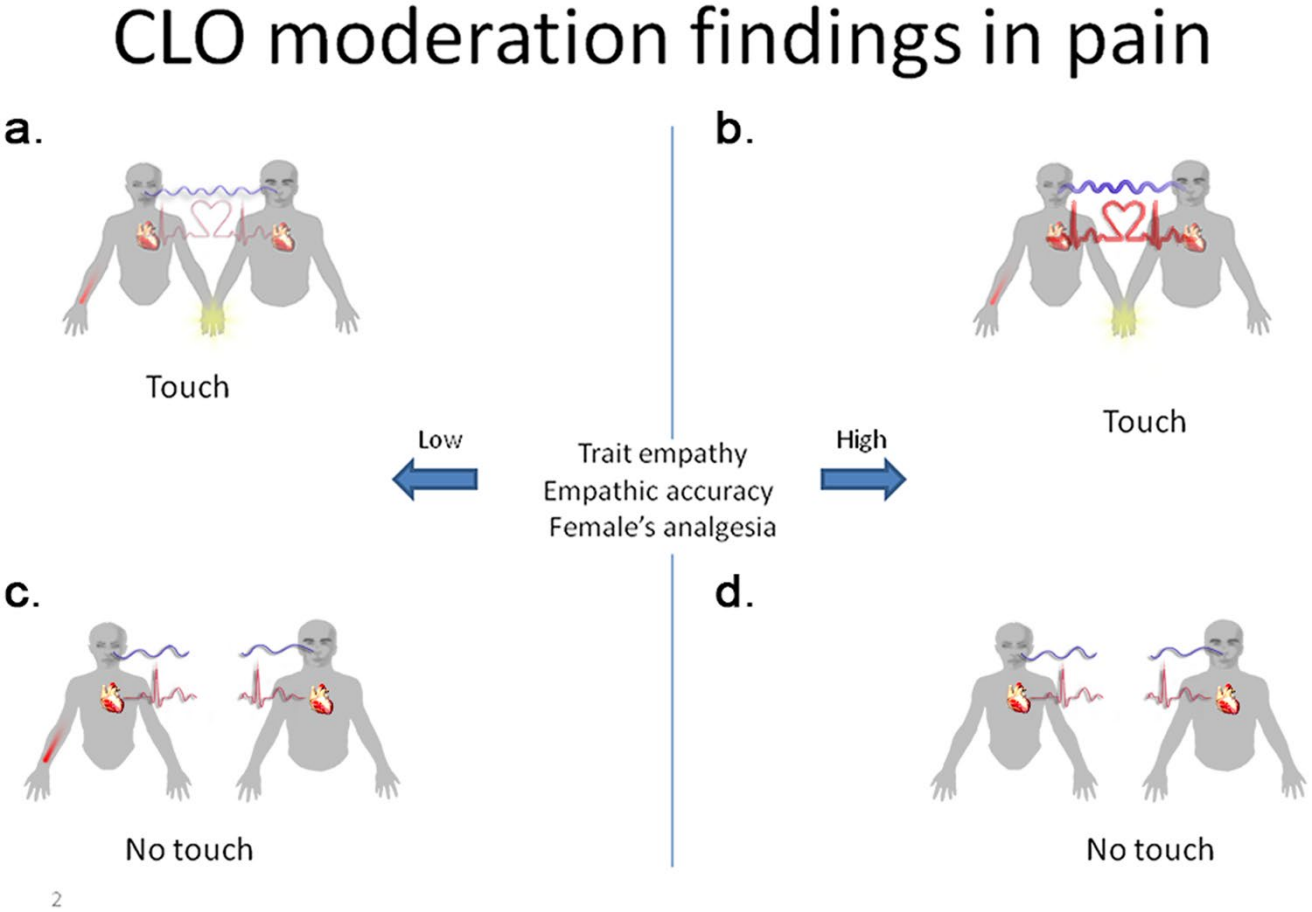
Graphical representation of moderation analysis of trait empathy, empathic accuracy and women’s analgesia on across-partner synchronization in HR and RR fluctuations. Empathic accuracy = man’s accuracy in estimating woman’s pain, trait empathy = IRI questionnaire, woman’s analgesia = reduction in woman’s pain as a result of man’s presence or touch. Blue and red lines mark respiration and heart rate inter-partner coupling, respectively. The line’s thickness represents the strength of the synchronization, and a broken line indicates a total lack of the coupling. (**a**) Coupling of respiration and heart rate during *touch-pain* condition for dyads with low (−1 SD) trait empathy, low empathic accuracy and low women’s analgesia. (**b**) Coupling of respiration and heart rate during *touch-pain* condition for dyads with high (+1 SD) trait empathy, high empathic accuracy and high women’s analgesia. (**c**) No coupling of respiration and heart rate during *no touch-pain* condition for dyads with low (−1 SD) trait empathy, low empathic accuracy and low women’s analgesia. (**d**) No coupling of respiration and heart rate during *no touch-pain* condition for dyads with high (+1 SD) trait empathy, high empathic accuracy and high women’s analgesia.

In line with our hypothesis, the male partner’s empathic accuracy significantly moderated the effect of touch on synchronization for respiration fluctuations (F_(4,28000)_ = 27.87, p < 0.0001, ΔBIC = 419439.8, ΔR^2^ = 0.23). More specifically, a high level of empathic accuracy (one standard deviation above the mean) compared to a low level (one standard deviation below the mean) was associated with increased coupling between female velocity and male acceleration in respiration during the touch condition (Δζ_t(p)_ = 0.028, p < 0.001, 95% CI [0.016, 0.040]). Correspondently, high levels of empathic accuracy between partners compared to low levels indicated increased coupling between male velocity and female acceleration in the touch condition (Δζ_t(p)_ = 0.029, p < 0.001, 95% CI [0.016, 0.041]) (Fig. 4a,b). The corresponding contrasts were not significant in the condition without partner touch (Δζ_nt(p)_ = 0.002, p = 0.56, men), (Δζ_nt(p)_ = 0.001, p < 0.73, women) (Fig. 4c,d).

We found significant moderation of the effect of women’s analgesia and touch on the cross-partner synchronization in velocity of respiration fluctuations (F_(4,28000)_ = 26.59, p < 0.0001, ΔBIC = −418367.9, ΔR^2^ = 0.19). Higher levels of women’s analgesia predicted increased coupling between female velocity and male acceleration (Δζ_t(p)_ = 0.027, p < 0.001, 95% CI [0.015, 0.039]) and increased associations between male velocity and female acceleration (Δζ_t(p)_ = 0.068, p < 0.001, 95% CI [0.042, 0.093]) in the touch condition. However, the corresponding contrasts were not significant in the absence of partner touch (Δζ_nt(p)_ = 0.005, p = 0.32, men; Δζ_nt(p)_ < 0.001, p = 0.87, women). It is important to note that trait empathy showed a pattern of moderation similar to that of empathic accuracy. However, the effect of trait empathy was redundant in the model that included empathic accuracy and women’s analgesia as moderators (most likely because of the high correlation between them).

The same pattern of moderation effects emerged in the heart rate analysis. Empathic accuracy and touch moderated the effect of synchronization in heart rate (F_(4,13000)_ = 20.73, p < 0.0001, ΔBIC = −265162.9, ΔR^2^ = 0.24). High as opposed to low levels of empathic accuracy predicted greater coupling between female velocity and male acceleration (Δζ_t(p)_ = 0.046, p < 0.001, 95% CI [0.026, 0.066]) and larger associations between male velocity and female acceleration (Δζ_t(p)_ = 0.049, p < 0.001, 95% CI [0.028, 0.070]) in the touch condition. The corresponding contrasts were not significant in the condition without partner touch (Δζ_nt(p)_ = 0.008, p = 0.28, males), (Δζ_nt(p)_ = 0.008, p = 0.34, females). As in the case of respiration fluctuations, women’s analgesia and touch significantly moderated the effect of cross-partner velocity for heart rate (F_(4,13000)_ = 5.03, p < 0.0001, ΔBIC = −267895.6, ΔR^2^ = 0.25). Specifically, high women’s analgesia as opposed to low analgesia was related to increased coupling between female velocity and male acceleration (Δζ_t(p)_ = 0.015, p < 0.001, 95% CI [0.008, 0.022]) and greater association between male velocity and female acceleration (Δζ_t(p)_ = 0.019, p < 0.001, 95% CI [0.010, 0.028]) in the touch condition. However, these contrasts were not significant in the no-touch conditions (Δζ_nt(p)_ = 0.003, p = 0.67, males), (Δζ_nt(p)_ = 0.022, p = 0.17, females). Similar to respiration fluctuations, trait empathy showed a pattern of moderation similar to that of empathic accuracy. However, the moderation effect of trait empathy did not contribute beyond empathic accuracy and women’s analgesia. In addition, the females’ feeling of comfort during the touch did not moderate coupling in respiration fluctuations (F_(4,28000)_ = 1.37, p = 0.24) nor heart rate (F_(4,13000)_ = 1.47, p = 0.21).

## Discussion

The current study sought to examine the role of touch and pain in inter-partner heart rate and respiration coupling.

The statistical analysis was based on the CLO model^20^, which allows estimating interpersonal bi-directional coupling between one partner’s signal exchange and a shift in the signal exchange in the interacting partner. Our findings confirm that interpersonal touch as compared to no-touch is associated with increased respiration coupling, during both pain and no-pain conditions. In line with these findings, interpersonal touch has been reported to increase coupling of electrodermal activity between partners^35^. Moreover, researchers have shown that touch can communicate emotions in a way that the receiver is able to recognize the emotional states communicated by the toucher^40–42^. Thus, in the current study the partners may have communicated their emotions via touch, as evidenced by an increase in physiological coupling.

The finding of an increased pattern of heart rate coupling during the pain and touch condition indicates that touch may allow communication between the participants but only during pain. This indicates that coupling in heart rate is evident only when the target experience pain and empathy in the observer is possibly provoked. Indeed, the powerful effect of social touch has been shown to affect our emotional well-being and diminish distress or pain in various settings^28, 37, 43–48^. For example, it has been reported that skin-to-skin touch may have an analgesic effect on human babies undergoing minor medical procedures^45^ and may even therapeutically reduce pain in cancer patients^44, 49^ and those with chronic pain^50^. Previously, Coan *et al*. (2006) reported that greater pain reduction is observed following the touch of a partner as compared to a stranger^37^. Thus, it could be suggested that inter-personal physiological coupling underlie touch-related analgesia.

In contrast to our original hypothesis, pain did not increase physiological coupling in the absence of the partner’s touch. This effect was consistent across both physiological signals and for both model directions (female-male, male-female). The decrease in coupling when the women experienced pain and their partners observed their pain partially contradicts the findings of Konvalinka *et al*.^17^. This study reports that during a fire-walking ritual, the cardiac rhythms of active participants were synchronized with those of related bystanders. However, one should take into account major differences between this study and the current study. While the sample studied by Konvalinka *et al*., 2011 consisted of experienced fire-walkers who were well trained to manage their pain and did not feel the pain^51^, our participants, who represented a normal population, did not have such experience. As a result they probably experienced higher levels of pain and distress during the pain condition, which may disrupt the physiological coupling. In addition, a possible explanation for these contradictory findings may rely on the different definitions of synchronization used in the two studies. Konvalinka (2011) defines synchronization as a similarity between the heart rate of a firewalker and that of a related spectator, while we defined interpersonal coupling as “one partner’s signal changing from increasing to decreasing while the other partner’s signal decreases”.

Here we show that in the absence of touch, the experience of pain disrupted the physiological coupling as the targets probably were focusing on their own pain experience. Indeed, previous research showed that pain interrupts the attention, that the suffering person pays to the external world^52^. It is thus possible that the women may have focused on her own pain and engaged in self-based strategies to cope with pain^53, 54^, which may explain their physiological “disconnection” from the male partners. In line with this idea, it was shown that social touch increases our attention to social stimuli^55^. However, self-based strategies seem to be less effective than those involving physical touch between partners, as we and others found that touch is associated with a greater analgesic effect than no-touch^36, 38^.

Indeed, the moderation analysis provides additional evidence for the notion that touch-related analgesia is related to the empathy of the toucher. The results show that both empathic accuracy and trait empathy moderated inter-partner physiological coupling, so that dyads with highly empathic male partners demonstrated increased coupling. That is, we observed a shift from exhalation to inhalation in a participant when their partner inhales (the heart rate shifts from a decrease to an increase when the partner exhibited an increase in heart rate).Consistent with this, researchers have shown that greater empathy is associated with better physiological linkage between romantic partners^56^ and greater touch-related coupling of electrodermal activity^35^. Moreover, in the couples where the female partners reported greater touch-related analgesia, we observed enhanced physiological coupling. Thus, the effects of touch on cardio-respiratory inter-partner coupling may be associated with pain analgesia. It is thus possible that cardio-respiratory changes, where the observer of pain affect’s the target of pain, is associated with the level of empathy of the observer. That is, the empathic response of the observer is communicated to the target of pain. This idea is in line with Hertestein *et al*. (2006) who demonstrated that people can identify various emotions including love and sympathy from the experience of being touched^41^.

Similarly, it is possible that the target of pain communicates back the analgesic effect of touch to the observer. Thus, the use of touch may improve the quality of non-verbal physiological communication between partners, especially when one of them feels pain, enabling the toucher to better project his empathy to the female partner and consequently have an analgesic effect.

It is important to note that the CLO model enables the identification of associations in both directions (i.e. the male partner signal change predicts the shift in signal change of the female partner and vice versa). However, in this study, both directions showed the same pattern of results supporting the idea of physiological bi-directional communication. These findings of physiological coupling may also explain recent findings of partners being able to influence women during labor. For example, it was reported that massage and breathing coaching from partners can decrease negative affect, as expressed by a depressed mood, anxiety, and pain as well as enhance positive affect, shorten labors and hospital stays, and decrease postpartum depression^57^.

Apparently, skin to skin touch is important for pain reduction, which may explain people’s preference for social touch^58^. Moreover, touch activates reward circuits in the brain^59, 60^. Indeed, skin-to-skin touch has been shown to activate the reward system, which results in pain reduction both in animals and in humans^47^. It seems that this phenomenon has evolutionary roots. For example, non-human primates devote much more time to grooming than they actually need for hygiene reasons, resulting in endogenous opioid release^61, 62^, as well as pain and stress reduction^63, 64^.

It is still not clear exactly how inter-personal physiological resonance is related to touch-related analgesia. Observing the pain of others can trigger emotional resonance in the observer, activating brain mechanisms similar to those of the suffering person (e.g., anterior cingulate and insula cortices) and areas that are classified as the “mirror neuron system” (e.g., inferior parietal cortex)^23^. As the parietal lobe integrates sensory information among various modalities, the assumption is that multisensory integration of both visual and tactile stimuli may facilitate the emotion resonance with the observed target, as also expressed in associated autonomic and somatic responses^65^. Moreover, tactile-induced analgesia^66^ correlates with activations in brain areas related to multimodal neural activity^67^ and emotional processes^68–72^. The partner’s touch may also enhance inter-partner brain synchronization in areas related to the pain matrix, a hypothesis that should be tested in future research using novel hyperscanning techniques^73^. In addition, the anterior cingulate cortex (ACC) has also been found to be relevant in the context of pain perception, empathy for pain^22^, touch^60^ and reward systems^74^. The ACC appears to play a role in a variety of autonomic functions, such as regulating heart rate and blood pressure^75, 76^. This complicated physiological mechanism may underlie the observed coupling between the partners.

Recent research shows that the neuropeptide oxytocin may play a key role in synchrony^77^ as well as in touch analgesia via physiological coupling. It was demonstrated that warm touch can increase the levels of plasma oxytocin, and reduce stress^78^ and depression^79^. Moreover, recent studies have highlighted the role of oxytocin in inter-personal coupling, increasing touch interaction synchronization while reducing stress^80, 81^ and enhancing brain-to brain coupling in alpha rhythms^82^. Thus, future research should test the role of oxytocin in touch-related analgesia.

It is important to note that our findings of interpersonal coupling during touch can also be explained by the phenomenon of Huygens synchronization of two connected pendulum clocks^83^. According to this explanation, the mere connection between objects creates synchrony between these objects. Nevertheless, the moderation effects of empathic accuracy and pain analgesia reduce the probability of this explanation. Interestingly, the difference in pain between the conditions with and without touch was higher in females than in males, who estimated their partners’ pain. These findings can be explained by the fact that female participants experienced the real pain and their male partners only guessed the females’ pain level. It is thus possible that males did not realize the magnitude of the effectiveness of their touch and therefore their pain ratings did not decrease in the touch condition.

Although the current study used a controlled design with several balanced conditions, it has several limitations that need to be acknowledged. First, only the female participants underwent pain stimuli while the male participants did not, so that the generalizability of our results is restricted to the male-to-female direction. Therefore, future research should test the effect of touch and pain in both men and women as well as in homosexual and heterosexual participants. This could also include parent-child, sibling, and best-friend interactions, in comparison to the interactions between strangers. Second, this study used a single subjectively adjusted degree of heat pain. Future research should test physiological coupling on varying pain intensities and also cold pain stimuli. Third, the subjects were asked for a static handholding, without squeezing, stroking or rubbing following the paradigm proposed by Goldstein *et al*. (2016). However, comparing between these different types of touch is of high interest and would benefit from future investigation. Fourth, partners could use visual information in all interacting conditions. The visual and tactile sources of information may interact in the brain and therefore future research should estimate these effects. Finally, reporting pain for both emotional and intensity components may shed more light on our findings.

To conclude, we show here that touch regulates physiological coupling during pain, suggesting that interpersonal coupling is affected by various contextual social cues. Yet the prevalent approach in testing perception and behavior is to split one complex system (e.g., the delivery of pain) into several subparts and to explore each of them independently. Although this simplified approach allows analyses of human responses, it lacks the sensitivity to capture elements involved in real-life social interactions. Considering that human behavior is fundamentally different when we are interacting with others rather than merely observing ourselves, here we investigate physiological response using a paradigm that also consider social contexts. Since physiological resonance has important evolutionary significance for animals and humans^84–86^, investigation of inter-personal coupling provides an interesting opportunity to understand our behavior in the natural social environment.

## Methods

### Participants

Twenty-two couples (44 participants) completed the study. Participants ranged in age from 23–32 years old (mean and SD for men: 26.4 ± 2.27 years; mean and SD for women: 25.6 ± 1.9 years), had no children, were in long-term relationships (mean and SD: 3.46 ± 2.25 years) and had around 13 years of education (mean and SD of years of education for men: 13.3 ± 1.5, and for women: 13.6 ± 1.3). Only 9% of the couples were married.

Since previous research indicated that women elicit more empathy and compassion^87^, men were always assigned the role of the observer, while women were the targets. Two of the couples were dropped from the analysis because of unsuccessful physiological recording.

The couples were screened by a phone interview based on the following criteria: (1) right-handed and between the ages of 22 and 40; (2) no chronic or acute pain of any sort; (3) no medication use (except for oral contraceptives); (4) no history of neurological disorders, psychiatric problems or other problems relevant to the study; (5) not pregnant; (6) in a romantic relationship (defined as couples who reported being in a serious relationship, living together for at least one year and having significant feelings of love for each other). The study was approved by the Faculty of Social Welfare and Health Sciences Ethics Committee, University of Haifa and informed consent was obtained from all participants. All methods were carried out in accordance with relevant guidelines and regulations.

### Assessment of empathy

Interpersonal Reactivity Index (IRI). The IRI is a 28-item questionnaire measuring empathic capacity on four separate subscales: (1) perspective taking; (2) empathic concern; (3) personal distress; (4) fantasy^88^. The reliability of all scales is 0.84. After the experiment terminated female participants were asked to rate: “How comfortable did you feel during the partner’s touch”? on a 1–10 scale.

### Pain familiarization and pain-60 determination

All contact heat stimuli in this experiment were applied to the left volar forearm using a 3 cm^2^ computer-controlled Peltier-type thermode (TSA-2001, Medoc, Ramat-Yishai, Israel) (see Fig. 1). During the procedure of pain familiarization, female participants were exposed to three short contact-heat stimuli (43, 45, and 47 °C), each for 7 seconds, administered in a semi-randomized order with a break of 10 seconds. Participants were asked to report pain intensity using the numerical pain score (NPS), ranging from 0, denoting “no pain” to 100, denoting “the worst pain imaginable.” Thereafter, the stimulus intensity was adjusted to each participant to evoke a peak pain magnitude of 60/100 (pain-60) on NPS, using the algorithm described by ref. 89.

### Experimental conditions

The experiment consisted of six experimental conditions within one session. The *pain-alone* condition included 120 seconds of pain stimulation applied to the woman’s left forearm at a temperature individually tailored to induce an NPS score of 60, while the partner sat in an adjacent room. During the * touch-no pain* condition, the participants sat facing each other holding their dominant hands, while during the *no touch-no pain* condition, the partner was only present without any physical contact. During the *touch-pain* condition, the pain stimulus was administered to the female participant while her partner held her dominant hand. In the *no touch-pain* condition, the female participant was administered the same pain stimulus, but her partner was only present without any physical contact. During the *no pain-alone* condition the partner sat in the adjacent rooms. In the no-touch conditions, participants were instructed to hold the handles of their armchair (Fig. 1). A 10 min break was kept between successive conditions.

#### Dual-ECG and respiration data acquisition

Standard electrocardiogram ECG readings were recorded simultaneously for both participants via MindWare MW1000A recorder (MindWare Technologies, Gahanna, OH) at a 256 Hz sampling rate, using Wi-Fi local network for the signal sync. Three ECG electrodes were placed on the right shoulder, and on the right and left lower quadrant correspondently. Respiration was recorded with respiration belts from MindWare Technologies (Gahanna, OH). The belt was placed around the waist just above the trousers. As a calibration procedure, participants were instructed to breathe at a normal rate into a bag of fixed volume (600 ml) for several cycles. This technology provides synchronous recording of all physiological signals from two participants.

### Procedure

Upon arrival, the partners were sent to different rooms and asked not to communicate verbally with each other until the experiment was over. After completing the IRI, participants underwent pain familiarization and pain-60 determination. This was followed by six counterbalanced conditions: *no pain-alone, pain-alone*, *partner touch-no pain, partner no touch-no pain, partner touch-pain* and *partner no touch-pain*. The women were asked to rate their pain intensity two seconds before the end of each condition using the NPS. Simultaneously, the male partners were instructed to rate their female partners’ level of pain. Both partners wrote the number on a small piece of paper not visible to the other member of the couple. A 10-minute break separated successive conditions.

### Pre-processing

Raw respiration data were cleaned from the clipping artifacts using an algorithm described by Helm *et al*.^20^. R-R intervals were calculated from raw ECG data by HRV 2.0 software (MindWare Technologies) and were then interpolated, as proposed by^90^, in order to merge the data between the partners.

### Statistical analysis

Our statistical framework was based on an entirely dyadic perspective that requires continuously measuring both individuals in the relationship at the same time. Examining physiological coupling between couple partners requires statistical models that can capture cross-partner dynamics. The model should take into account the trajectory of a given physiological signal and to estimate the association between the couple partners. Since respiration and heart rate follow oscillating patterns, the model should refer to the sinusoidal fluctuating pattern of the signal, testing the relationship between the partners’ signals. Linear oscillator (CLO) model^20^, which is an extension of the Damped Linear Oscillator model (DLO)^91^ suits these requirements, providing a complex framework for the given type of the data. The DLO model uses estimates of the first derivative (signal change or velocity) and the second derivative (shift in signal change or acceleration) of a dynamic signal to model data that fluctuate around a constant point over time. The parameter η in the DLO describes the relationship between the position (signal itself) and the acceleration, while the parameter ζ quantifies the relationship between the velocity and the acceleration. The CLO model is the bivariate extension of the DLO and provides an estimation of linkage between two members of a system (for all details see ref. 20). The model can be characterized by the following equations:

$$\frac{{d}^{2}{x}_{(t)}}{d{t}^{2}}=\frac{{{\rm{\zeta }}}_{{\rm{s}}}d{x}_{(t)}}{dt}+{{\rm{\eta }}}_{{\rm{s}}}d{x}_{(t)}+\frac{{{\rm{\zeta }}}_{{\rm{p}}}d{y}_{(t)}}{dt}+{{\rm{\eta }}}_{{\rm{p}}}d{y}_{(t)}\,+{\varepsilon }_{x(t)},\,and$$

$$\frac{{d}^{2}{y}_{(t)}}{d{t}^{2}}=\frac{{{\rm{\zeta }}}_{{\rm{s}}}d{y}_{(t)}}{dt}+{{\rm{\eta }}}_{{\rm{s}}}d{y}_{(t)}+\frac{{{\rm{\zeta }}}_{{\rm{p}}}d{x}_{(t)}}{dt}+{{\rm{\eta }}}_{{\rm{p}}}d{x}_{(t)}\,+{\varepsilon }_{y(t)}$$

where x and y are physiological signals from the two members of the dyad. The first two terms of each equation characterize within-person linkage between velocity and position, with acceleration as defined in DLO model. The next two terms describe corresponding cross-partner associations that can be interpreted as inter-personal coupling. In this study, we refer to inter-partner velocity-acceleration coupling in heart rate and respiration as an indicator of interpersonal synchronization. Positive ${{\rm{\zeta }}}_{{\rm{p}}}$ can be interpreted as changes in one partner’s signal from decreasing to increasing while the other partner’s signal increases.

To analyze the data, we used Linear Mixed Models (LMM) via MIXED procedure in SAS^92^. LMM allows taking the hierarchical structure of data into account^93^. The Bayesian Information Criterion (BIC) was used for model selection. The model with the smaller BIC shows a better fit. Since a slope representing signal trends is absent in the CLO model, all data were linearly detrended^94^.

To estimate the partners’ velocity effect in each condition, we applied post-hoc contrast analysis using simulation-based multiple test correction proposed by^95^. Moderation analysis of empathy and pain reduction on cross-partner coupling was conducted in the same statistical framework, including corresponding interaction terms.

In order to systemize reporting of the findings, we used the following condition notations: “p” = pain, “np” = no pain, “t” = touch, “nt” = no touch, “a’ = alone. Thus, ζ_nt(np)_ means coupling during the *no touch-no pain* condition.

Empathic Accuracy: The measure of empathic accuracy was defined as the absolute difference between the partners’ pain ratings (each male partner’s pain ratings minus his female partner’s pain ratings) divided by the female partner’s pain ratings. Touch related analgesia: We also referred to reduction in the female partner’s pain (i.e., women’s analgesia) and calculated the percentage difference between each woman’s pain rating in the *no touch-pain* and the *touch-pain* conditions and her rating in the *pain-alone* condition. In the moderation analysis the three-way interaction term of partner’s velocity X touch X moderator was tested and then corresponding post-hoc contrast analysis was applied to interpret the findings.
